# Targeting the mTOR Signaling Pathway Utilizing Nanoparticles: A Critical Overview

**DOI:** 10.3390/cancers11010082

**Published:** 2019-01-11

**Authors:** Mariia Lunova, Barbora Smolková, Anna Lynnyk, Mariia Uzhytchak, Milan Jirsa, Šárka Kubinová, Alexandr Dejneka, Oleg Lunov

**Affiliations:** 1 Institute of Physics of the Czech Academy of Sciences, Prague 18221, Czech Republic; mariialunova@googlemail.com (M.L.); smolkova@fzu.cz (B.S.); lynnyk@fzu.cz (A.L.); uzhytchak@fzu.cz (M.U.); sarka.k@biomed.cas.cz (Š.K.); dejneka@fzu.cz (A.D.); 2 Institute for Clinical & Experimental Medicine (IKEM), Prague 14021, Czech Republic; miji@ikem.cz; 3 Institute of Experimental Medicine of the Czech Academy of Sciences, Prague 14220, Czech Republic

**Keywords:** lysosomes, molecular targeting, nanoparticles, mTOR, reactive oxygen species

## Abstract

Proteins of the mammalian target of rapamycin (mTOR) signaling axis are overexpressed or mutated in cancers. However, clinical inhibition of mTOR signaling as a therapeutic strategy in oncology shows rather limited progress. Nanoparticle-based mTOR targeted therapy proposes an attractive therapeutic option for various types of cancers. Along with the progress in the biomedical applications of nanoparticles, we start to realize the challenges and opportunities that lie ahead. Here, we critically analyze the current literature on the modulation of mTOR activity by nanoparticles, demonstrate the complexity of cellular responses to functionalized nanoparticles, and underline challenges lying in the identification of the molecular mechanisms of mTOR signaling affected by nanoparticles. We propose the idea that subcytotoxic doses of nanoparticles could be relevant for the induction of subcellular structural changes with possible involvement of mTORC1 signaling. The evaluation of the mechanisms and therapeutic effects of nanoparticle-based mTOR modulation will provide fundamental knowledge which could help in developing safe and efficient nano-therapeutics.

## 1. Introduction

Clinical applications of nanoparticles (NPs) and nanotechnology are rapidly growing. Because of their unique size-dependent properties, NPs are becoming indispensable as material coatings, probes for cell and cell structures labeling, cancer treatment, as well as means of drug and gene delivery [1,2,3,4,5]. Chemical and physical properties that cannot be achieved by bulk materials represent the core of NPs uniqueness [6]. The integration of nanotechnology with pharmaceutical and biomedical sciences resulted in the appearance of the novel field of nanomedicine that aims to develop nanoparticle-based medicines with higher efficacy and improved safety and toxicological profiles [7,8]. Currently, there are 51 FDA-approved nanomedicines and 77 products in clinical trials [7]. The possibility to use NPs for selective detection and killing of cancer cells still remains up to date and intriguing [7,8,9,10]. Moreover, there is an urgent need for the development of novel therapies because conventional cancer therapies are not that effective due to their intrinsic limitations [11,12].

Nano-research has generated a myriad of different NPs possessing distinct physicochemical properties (e.g., size, shape, core composition, shell thickness, and surface chemistry) and having multiple biological functions [13,14]. Indeed, several nanomedicine platforms have already shown great promise in clinical studies [8].

The serine/threonine kinase mammalian target of rapamycin (mTOR) is a key kinase that controls cell growth and proliferation under favorable environmental conditions, integrating diverse environmental cues (nutritional and hormone/growth factor-mediated) [15,16]. A number of cancers overexpress or possess mutated forms of mTOR and of some of the targets of the mTOR kinase signaling [17,18]. Thus, mTOR signaling has been recognized as a promising target for anticancer treatment [17,18,19]. mTOR inhibitors have shown convenient pharmacological profiles and are well tolerated compared to conventional anticancer drugs [19,20,21]. It is worth noting here that various NPs have demonstrated successful ability to modulate mTOR activity [22,23,24,25]. For instance, amino-decorated NPs steadily inhibited mTOR activity and proliferation in three leukemia cell lines [23]. However, the current knowledge of the physiological and pathophysiological effects of NPs on cancer cells remains modest.

Only recently, reports started to challenge the applicability of cancer nanomedicine, arguing that translation of the laboratory results to successful clinical applications is very limited [26,27]. A highly innovative study has shown that nanomedicine delivery efficiency of about 0.7% of injected NPs to solid tumors is not superior to that of conventional drugs [27]. This study unfolded a debate over the clinical translation of nanomedicine [28,29]. The same study also unraveled other challenges of the clinical applicability of NPs including interactions and fate of nanomedicines in tumors [27,30]. Despite the enormous progress in the field of cancer nanomedicine, the literature lacks sufficient studies on the evaluation of intratumoral kinetics, interactions, and fate of nanomedicines [9,27,30]. It has been recognized that the modulation of mTOR could be a hint that underlies the biological effects of engineered NPs [31]. However, understanding the mechanisms of NP-mediated mTOR modulation is in its infant state.

In this review, we aim to provide an overview of recent investigations on NP-mediated cell signaling focused on mTOR modulation and identify gaps in our understanding of mTOR signal modulation by NPs. Lysosomal stability has been considered a mediator of nanoparticle signaling to the mTOR cascade [23,31,32,33]. The so-called “proton sponge effect” was originally postulated as the main factor responsible for lysosomal stability or impairment by NPs [13,32]. However, novel findings question the “proton sponge effect” as the dominant mechanism of lysosomal stability [34,35]. Here, we provide a comprehensive account of the involvement of the “proton sponge effect” in lysosomal modulation triggered by NPs. Moreover, we provide our vision of the challenges in the identification of the molecular mechanisms of mTOR signaling modulation by NPs and the resulting cellular processes.

## 2. Mammalian Target of Rapamycin Signaling as a Pharmacologic Target

The mechanistic/mammalian target of rapamycin (mTOR), also known as FK506-binding protein 12-rapamycin-associated protein 1, is the key regulator of cell metabolism homeostasis [17,36]. mTOR regulates multiple intracellular processes ranging from cell growth and proliferation to distinct death pathways [17,36]. A single gene encodes mTOR in mammals [17]. It is well established that mTOR interacts with several proteins to form two distinct complexes referred to as mTORC1 and mTORC2. Various signals elicit rapamycin-sensitive mTORC1 complex responses. Activated mTORC1 switches cell metabolism from the catabolic to the anabolic program. Such switching promotes protein synthesis and cell growth while repressing autophagy [15,16,17,18]. Indeed, cell growth and proliferation are positively regulated by mTORC1 via activation of many anabolic processes, including biosynthesis of proteins, lipids, and organelles, and by limiting catabolic processes such as autophagy [37].

**Figure 1:**
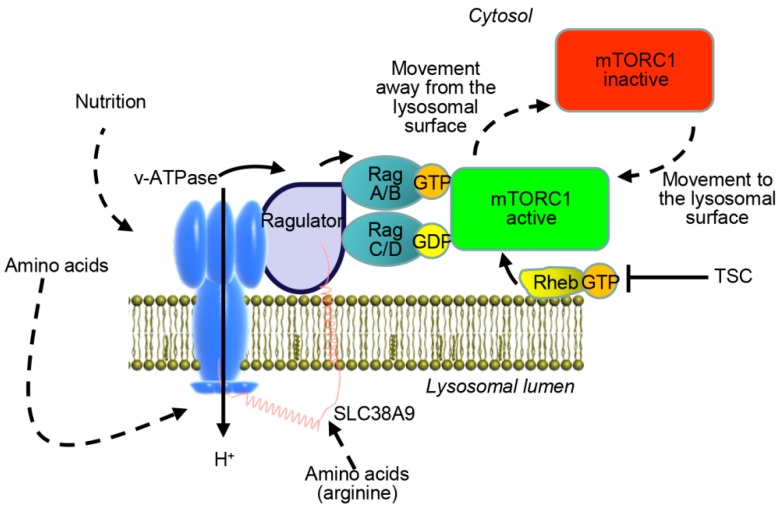
Mammalian target of rapamycin (mTOR) signaling at the lysosomal surface. Under growth-promoting conditions, Rag (Ras-related GTPases) and Rheb (Ras homolog enriched in brain) GTPases activities result in the recruitment and activation of the mTORC1 complex. Loss of these inputs leads to blockade of mTORC1. TSC: Tuberous sclerosis complex; v-ATPase: vacuolar-type H^+^-ATPase.

It has become widely accepted that the lysosomal membrane is the major site for mTORC1 activation [17,18,38,39,40]. A detailed mechanism of mTOR activation at the lysosomal site has been reviewed previously [41,42]. In fact, growth factors, energy status, oxygen, and amino acids are major signals that are integrated by mTORC1 [37]. The tuberous sclerosis complex (TSC) represents one of the most important sensors involved in the regulation of mTORC1 activity [17,18,38,39,40]. TSC was shown to inhibit mTORC1 in response to endogenous reactive oxygen species (ROS) [43,44]. Conversely, TSC blockade results in the activation of the small Ras-related GTPase Rheb (Ras homolog enriched in brain). The active form of Rheb then directly interacts with mTORC1 to stimulate its activity [37]. Overall, mTOR signaling has been addressed previously in a very comprehensive, well-structured, and illustrated review [37]. Here, we briefly summarize mTOR activation in Figure 1.

Upon stimulation by growth factors such as insulin, the serine/threonine kinase AKT is activated and phosphorylates TSC [37,41]. This phosphorylation results in the dissociation of TSC from the lysosomes, where TSC resides in growth factor-deprived conditions, and thus blocks its inhibitory effects toward Rheb [45,46]. mTORC1 is recruited to the lysosomal membrane from the cytosol under normal (non-stressed), nutrition-replete conditions. The recruitment to and retention of mTORC1 in the lysosomes is regulated through an amino acid-sensing cascade involving v-ATPase (vacuolar-type H^+^-ATPase), Ragulator, and Rag (Ras-related GTPases) GTPases [17,18,38,39,40]. v-ATPase supports Ragulator activation which leads to the formation of active heterodimeric complexes RagA/B–RagC/D in the lysosomes [17,18,38,39,40,47]. The Rag GTPase complex becomes active by acquiring an activated guanyl-loaded configuration in which RagA/B is GTP-loaded and RagC/D is GDP-loaded [38,40]. Such configuration facilitates the lysosomal attachment of mTORC1 by direct interaction with Raptor [38,40]. Active Rag GTPases translocate mTORC1 to the lysosomes where the kinase is activated by Rheb [17,18,38,39,40,47]. The activation of Rag GTPases is mediated by the presence of amino acids [17]. Recent studies advocate SLC38A9 (a putative sodium-coupled amino acid transporter in the lysosome membrane) to be a sensor that signals arginine sufficiency to mTORC1 [48,49,50].

Other lysosomal amino acid transporters such as SLC15A4 [51] and proton-assisted amino acid transporter 1 (PAT1)/SLC36A1 [52] have also been shown to be involved in mTORC1 activation. The latter transporter functions as a symporter of amino acids with protons in stoichiometry 1:1 [52]. Therefore, for the adequate transport of amino acids, availability of free protons is required. This implies that a functional v-ATPase and low pH in the lysosomal lumen are needed for successful mTORC1 activation. Whereas reduced lysosomal function due to v-ATPase inhibition resulted in strong mTORC1 inactivation with subsequent reduction of mTOR-dependent phosphorylation [53,54,55], mounting evidence suggests that activation of lysosomal function (acidification and delivery of hydrolases) is associated with suppression of mTOR activity [56,57,58]. It is noteworthy that, in some specific cell types such as chondrocytes, pharmacological inhibition of lysosomal acidification activates the mTORC1 signaling pathway [59].

Mammalian target of rapamycin is a well-accepted negative regulator of autophagy [17,36], and low lysosomal pH is crucial for the successful execution of autophagy [60,61]. Autophagy is a self-degradative process by which cytoplasmic materials are delivered to and degraded in the lysosomes [62]. Increased lysosomal acidification and enhanced autophagic flux then inhibits mTORC1 signaling [63].

Summarizing all these data together is puzzling. On one hand, v-ATPase integrity and low pH in the lysosomes are crucial for mTORC1 activation [53]. On the other hand, it was shown that lysosomal acidification is reduced in MCF-7 cells [63]. Additionally, those cells exhibit increased levels of autophagosomes and increased activity of mTORC1 [63]. How can that be explained?

Lysosomotropic fluorescent dyes are used to analyze lysosomal activity but do not provide quantitative measurements of lysosomal pH. None of the studies assessed the extent of lysosomal pH changes; nonetheless, there is emerging evidence showing that the autolysosome possesses even lower pH than a lysosome upon fusion with an autophagosome [56]. We propose the tentative following description. In favorable environmental conditions, a “normal” state of lysosomal acidification persists, which includes functional v-ATPase and low pH (≈4.6–5.6) of the lysosomal compartments. This state promotes mTORC1 activity, cell growth, and proliferation. “Decreased” acidification (pH > 5.6) is most likely responsible for the inhibition of mTOR signaling, cell cycle arrest, and cell death. Contrarily, “increased” acidification (pH < 4.6) accompanies the autophagic process that suppresses mTORC1. Thus, the lysosomal pH should be tightly regulated to preserve normal mTOR function. Indeed, it is not surprising that altered v-ATPase activity and lysosomal pH dysregulation together with altered mTOR signaling have been found in various pathophysiological conditions [17,36,64].

Deregulated mTOR signaling was found in ageing and human diseases such as cancer and metabolic diseases [15,17]. Whereas dysfunction of autophagy that promotes cancer through cellular stress and the role of mTOR in this process are discussed by Paquette et al. [65], oncogenic signal transduction through mTOR is deliberately discussed by Rad et al. [66]. Due to the prevalence of mTORC1 activation in human cancers, there is a growing interest in mTORC1 inhibitors for the treatment of a wide variety of cancers, including solid carcinomas and sarcomas, as well as those of hematopoietic origins [67]. Thus, mTOR as well as some of the targets of mTOR kinase are recognized as promising pharmacological targets [17,18,19]. Indeed, rapamycin showed excellent anticancer properties in vitro [19]. However, to date, the application of rapamycin as an anti-cancer drug in clinical trials has shown limited success [17,19]. Thus, many derivatives of rapamycin, known as rapalogs (e.g., temsirolimus, everolimus46, ridaforolimus), were developed to improve the pharmacokinetic properties and efficacy of rapamycin [68,69]. Despite the promise of rapalogs, they have achieved modest effects in treating major solid tumors. The reasons for the limited clinical success of rapalogs have not been established [69]. It is worth noting that recent studies have demonstrated that novel v-ATPase inhibitors, which have inhibition selectivity, can be systemically administered to animals and are highly efficacious against different cancer models in vivo [70,71,72]. However, so far, there is only preclinical evidence that v-ATPase inhibitors can enhance the efficacy of many cancer therapies [70,71,72]. Further clinical trials are needed to provide solid proof for the use of v-ATPase inhibitors in the treatment of cancers.

Mammalian target of rapamycin kinase inhibitors are under development and being tested for their impact on autophagy regulation [15], tissue hypertrophy [73], diabetes, and ageing [17], and for treatment of different cancers [74,75]. At this point, the anti-angiogenic properties of mTOR inhibitors have shown their potential in various cancer models (reviewed by Faes et al. [76]). However, despite a significant efficacy in pre-clinical models, the clinical tumor response to mTOR inhibitors is relatively modest [19,77] because the compounds have only limited efficacy as single agents in cancer therapy [75]. There are several factors which might explain this limited impact in clinical applications [69]: Incomplete inhibition of mTORC1 [78], mutations in mTOR causing its resistance to inhibitors, activation of alternate proliferative signaling pathways, and intratumoral heterogeneity of mTOR activity [79]. Although the performance of the tested mTOR inhibitors in cancer therapy was moderate, it is still believed that the full therapeutic potential of targeting mTOR has yet to be exploited [19,77], and NP-based medicines are thought to have the ability to overcome the problems presented by regular drugs in mTOR signaling modulation.

## 3. Key Examples of Nanomaterials Used in Drug Products

In recent years, we faced a rapid development and expansion of the field of nanomedicine [80,81,82]. Indeed, nanomedicine is a relatively new and rapidly evolving field that merges nanotechnology with the biomedical and pharmaceutical sciences. It is worth mentioning here that the development and application of nanotechnology in medicine has resulted in significant advances in the diagnosis, treatment, and prevention of different diseases [8,80,81,82]. Nowadays, we define NP-based medicines as drugs or biologically active compounds that incorporate NPs (1–100 nm) in order to achieve beneficial biomedical applications [7]. These applications lead to improved targeting, reduced toxicity, or otherwise enhanced efficacy of therapeutic or imaging agents in vivo [7]. Nanoparticles have unique biological properties due to their sub-micrometer size and high surface-area-to-volume ratio [80]. In fact, NPs show key differences in comparison to bulk materials, including specific biochemical, magnetic, optical, and electronic properties [14,80,83,84]. Additionally, NPs’ large functional surface area allows them to bind, absorb, and carry other compounds such as small-molecule drugs, DNA, RNA, proteins, and probes [82]. Therefore, the unique physicochemical and biological properties of NPs have allowed the generation of several NP-based medicines, such as liposomes, albumin NPs, and polymeric micelles, that have been approved for cancer treatment [7,8,80,82]. Moreover, there are many other nanotechnology-based therapies that are currently under clinical investigation, including chemotherapy, hyperthermia, radiation therapy, gene or RNA interference therapy, and immunotherapy [8,82]. A recent study has identified 51 FDA-approved NP-based medicines and 77 products in clinical trials [7]. NPs used in nanodrug formulations currently include liposomes, polymers, micelles, nanocrystals, metals/metal oxides and other inorganic materials, and proteins, although research is also being conducted with other types of NPs, such as carbon nanotubes (Table 1).

It is worth noting here that the tunable size, shape, and surface characteristics of NPs allow them to have high stability, high carrier capacity, and the ability to incorporate both hydrophilic and hydrophobic substances [80,81,82]. Such characteristics of NPs make them compatible with different administration routes. The major routes of administration of drug products containing nanomaterials are the following: Intravenous, oral, ophthalmic, inhalation (oral/nasal), topical (skin), intramuscular, and vaginal. Indeed, a majority of NP-based medicines typically use intravenous administration. Oral administration is a second commonly utilized type of nanoparticle administration [7,8,80,82,85]. A recent comprehensive analysis showed that most NP-based medicines focus on cancer treatment (35%), followed by inflammatory/immune/pain disorders (18%) and infections (12%) [80].

Targeted delivery is one of the highly researched areas of nanotechnology. It was postulated that NP-based targeting would revolutionize the treatment of cancer [82]. Nonspecific biodistribution and persistent background retention dramatically affect the target efficiency of NPs [86]. Indeed, there are passive and active targeting strategies or a combination of both [87]. Passive targeting utilizes the so-called enhanced permeation and retention (EPR) effect. Generally, the EPR effect is referred to as the phenomenon by which NPs tend to accumulate preferentially in tumor tissues because of the leaky tumor vasculature and poor lymphatic drainage [8]. Active targeting relies on the specific interactions between targeting ligands on the particles and markers associated with the tumor. Such targeting strategy results in enhanced accumulation or retention of particles at the tumor site or in increased uptake of particles by cells expressing the target receptor [8,82]. To extend the tumor retention of small molecules, an effective strategy is to conjugate active targeting ligands (e.g., proteins, peptides, aptamers) or to use a mechanism of selective tumor uptake (the most successful examples is (^18^F)FDG, i.e., glucose labeled with radioactive ^18^F) [88,89,90]. By utilizing specific molecular motifs, one can target specific cell types (for reviews see [88,89,90]). Through passive tumor targeting via the EPR effect, one can get a tumor targeting efficiency higher than 7% of the injected dose (ID)/g for various types of NPs in different tumor xenograft mouse models, such as EMT-6 (mouse breast carcinoma), MDA-MB-435 (human melanoma), U-87 MG (human glioblastoma), and 4T1 (mouse breast carcinoma) (for review see [88]). Polyethylene glycol (PEG)ylated Au NPS have been shown to actively accumulate in tumors with targeting efficiencies of 15.3% ID/g [91] and 12.5% ID/g [92].

## 4. Examples of Side Effects of Nanomaterials

Generally, NPs have been successfully utilized to reduce their free-drug counterparts’ toxicity and improve drug accumulation at the site of action [7,8,80,82,85]. However, despite their beneficial impacts, the use of nano-based drugs raises several safety concerns.

Cytotoxic effects in vitro as well as in vivo have been reported more regularly with some categories of NPs [13,93,94,95,96,97]. Frequently, nanoparticle-induced cytotoxicity is caused by lipid membranes damage and impairment of cell homeostasis [13,93,94,95,96,97]. Recent studies showed lysosomal membrane degradation after exposure to different NPs types, such as polystyrene [98], titanium dioxide [99], and zinc oxide [100]. Additionally, lysosomal leakage together with the release of lysosomal contents may result in mitochondrial and endoplasmic reticulum dysfunction [13,93,94,95,96,97]. In fact, lysosomal dysfunction leads to ROS production, which results in oxidative stress [13,93,94,95,96,97].

The undesired immune system activation represents one of the major limitations for the successful clinical use of NP-based medicines [101,102,103]. Indeed, cells of the mononuclear phagocytic system, especially phagocytic macrophages, are able to recognize and engulf NPs [101,102,103]. This process begins with opsonization, together with absorption of plasma proteins (including serum albumin, apolipoproteins, immunoglobulins, and components of the complement system), onto the surface of NPs [13,104]. After protein absorption, NPs are attached to specific surface receptors of phagocytes, then internalized and transported to the lysosomes [105,106]. Additionally, some NPs can directly stimulate the immune system by binding to Toll-like receptors and, together with complement activation, enhance the inflammatory response [107]. Moreover, clearance of NPs by macrophages reduces their accumulation in the target site and subsequently decreases the therapeutic efficiency [26,27,101,102,103]. Nowadays, several approaches have been suggested to overcome NP-directed immune response [108,109,110]. The most common way is the usage of antifouling agents to decrease protein binding. One of the best examples is PEG [26,27,101,102,103]. However, coating of NPs with PEG does not reduce protein binding completely [109]. Another strategy utilizes the binding of lipoproteins (high-density lipoprotein and low-density lipoprotein), which are able to prevent complement activation [110]. Indeed, the inhibition of distinct components of the complement systems seems to be very effective in reducing nanoparticle-induced immune response [108].

Moreover, there is a still persistent problem with targeted delivery of NPs. The EPR effect is typically believed to be responsible for increased delivery of NPs to targeted tumors in animal experiments [8,26,27,88]. However, very often, the interpretation of EPR is oversimplified and overestimated [8,26,27]. A careful analysis of the nanoparticle delivery literature from the past decade revealed that the median delivery efficiency of NPs is still low (only 0.7% of an injected dose) [27]. Only isolated studies show delivery efficiency >7% of the injected dose (for review see [88]). On a large scale, this effect shrinks to ≈0.7% ID [27]. This has negative consequences for the translation of nanotechnology for human use in clinical applications [27].

A conceptual understanding of the biological responses to nanomaterials and the consideration of their side effects are needed to develop and apply safe nanomedicines.

## 5. Nanoparticles in the Modulation of Mammalian Target of Rapamycin Activity: Challenges in Finding Mechanisms

An increasing number of publications in recent years suggest that various NPs modulate mTOR activation, leading to cell cycle arrest in cancer cells [22,23,24,25]. The regulation of cell death/survival and metabolic responses by NPs via modulation of mTOR was postulated previously [31]. However, the current knowledge of the possible mechanisms that drive mTOR-related effects of NPs on cells remains limited. We have summarized the current literature about mTOR signaling modulation by engineered NPs in Table 2. 

Depending on their composition and original source, NPs can be divided into two groups. Natural-based NPs are typically synthesized from biomolecules, particularly chitosan, lactic acid, dextran, lipids, and phospholipids. Chemical-based NPs are frequently made from synthetic materials such as metals, silica, various polymers, and carbon. Rapamycin-loaded liposome formulations were proposed as an efficient alternative compared to the free-drug composition for therapy of breast cancer [124]. The advantages of rapamycin liposome formulations could be potentially explained by their stability, fluidity, proper drug distribution and incorporation, and loading of rapamycin into the lipid bilayer [125].

Another formulation that could be recognized as nano-based is polyamidoamine (PAMAM) dendrimer [126]. PAMAM NPs deregulate mTOR and its downstream signaling pathway and induce autophagic cell death [116,127].

It is also possible to create protein-based NPs. Utilizing albumin-bound rapamycin NPs, one can effectively increase the lifespan of an animal xenograft model of multiple myeloma [128].

Further, the PI3K–Akt–mTOR signaling pathway could be inhibited by SiO_2_ NPs [129]. Mechanistically, SiO_2_ NPs deregulate the NO–NOS system and trigger an inflammatory response which results in autophagy [129]. Amino-functionalized polystyrene nanoparticle treatment initiates G2 cell cycle arrest and blocks proliferation and vascularization in leukemia cell lines through the inhibition of mTOR signaling pathways [23]. Furthermore, COOH-functionalized carbon nanotubes exert a dramatic autophagic effect on the cells through modulation of the AKT–TSC2–mTOR pathway [25].

In summary, the majority of NPs resulted in mTOR inhibition in various types of both cancer and normal cells (Table 2). However, if one tries to find out any pattern in NP–mTOR relations, the data are lacking. We actually see that NPs of different chemical composition, size, and shape are able to affect mTOR signaling, and it is difficult to decipher any pattern in their chemical and biophysical effects.

NPs may affect lysosomal recruitment and activation of mTORC1 via interaction with lysosomes [23,33]. What initiates this recruitment is not known. Alternatively, NPs may affect mTOR signaling via ROS production [22,23,24,111,121]. ROS accumulation has been linked with the stimulation or suppression of mTORC1 activity [130]. However, this cannot explain the bewildering effects of NPs on mTOR activity, taking into account that ROS production and accumulation initiated by NPs starts from lysosomes [13]. Another popular explanation is the “proton sponge” effect or “proton sponge” hypothesis [13,31,34]. According to this hypothesis, positively charged (e.g., amino-functionalized or polyallylamine hydrochloride-coated) polystyrene, poly-amine dendrimer, or rare-earth upconversion NPs induce lysosomal swelling and damage via osmotic destabilization [13,34]. Unprotonated amines of positively charged particles/polymers can absorb protons as they are pumped into the lysosomes, resulting in more protons being pumped. This leads to an increased influx of Cl^−^ ions and water. Left uncontrolled, this process causes lysosomal swelling and rupture [13,34]. Subsequently, such osmotic disturbance causes lysosomal dysfunction and inhibition of mTORC1 [23,31]. In general, the “proton sponge” hypothesis was adopted from the proposed mechanistic understanding of the interaction between non-viral vectors made of, coated with, or just containing polycations, such as polyethylenimine (PEI), and cells [34]. However, the “proton sponge” hypothesis is contradicted by recent research showing that buffering polymers are unable to increase the endolysosomal pH [34,35]. These data clearly show that the osmotic effect alone is perhaps insufficient to induce lysosomal leakage or rupture [34,35]. v-ATPase is capable of overcoming the “proton sponge” effect and stabilize the pH [35]. In regard to NPs, even some negatively charged NPs inhibited mTOR via lysosomal dysfunction (Table 1). Thus, this hints at the involvement of additional factors other than surface charge/chemical functionalization that contribute to the modulation of mTOR activity by NPs.

In biological environments, NPs are not “naked”. They are covered with a layer of biomolecules, predominantly proteins [131,132]. This so-called protein corona forms around NPs in protein-rich fluids found to be crucial in mediating subsequent NP-triggered interactions with cells [131,132,133]. Blood circulation, extravasation into and interaction with the perivascular tissue microenvironment, tissue penetration, and cell internalization are influenced by the formation of the protein corona [131,132,133]. The protein corona can also give rise to undesirable adverse effects, e.g., the loss of NPs’ targeting capabilities [134]. For example, the protein corona may reduce nanoparticle cell membrane adhesion, mitigating the disruption of cell membranes by bare NPs [135]. The proteins adsorbed on NPs cause a loss or reduction of the targeting capability of surface-functionalized NPs [134]. In addition, adsorbed proteins undergo conformational changes on the surface of NPs [136,137,138]. Such conformational changes can modify cell recognition by NPs and initiate alternative cell signal transduction [136,137,138,139]. In addition, the adsorbed proteins may support mTOR activation and negatively impact on the specificity of NPs to induce apoptosis in cancer cells [33]. Indeed, structural changes induced by NPs have been reported for ribonuclease A, cytochrome c, albumin [140,141,142]. Additionally, NPs can induce protein aggregation [143]. This can trigger an immune response and affect NPs’ toxicity and targeting capabilities [144,145].

Nanoparticles are typically internalized into cells where they are trafficked along the well-defined endo-lysosomal pathway [13]. After engulfment by a cell, nanoparticles accumulate in acidic vesicular organelles, such as endosomes and lysosomes [1,13,146]. Endocytosed nanomaterials are degraded by hydrolytic enzymes abundant in these organelles. Specific proteins present in the original protein corona are retained on the NPs until they accumulate in lysosomes [132]. The protein layer may play a crucial role in triggering distinct cellular functions [131,132]. In fact, the power of nanoparticles to carry proteins that are atypical in endogenous processes has many potential implications [132]. These processes have not been investigated in detail. Therefore, until now, we poorly understand the phenomena of intracellular nanoparticle-mediated protein trafficking [132]. Generally, protein coronas reduce the cytotoxicity and immunotoxicity of NPs [144,145,147]. However, sometimes, cytotoxicity and immunotoxicity can be mitigated or activated depending on the type of NP and adsorbed plasma proteins [144,145,147]. The physicochemical surface properties of NPs (ie, physical surface architecture and chemical functionality) influence the immunological response to NPs’ protein coronas [147]. Therefore, future works should investigate the effective physicochemical properties of NPs to determine their protein coronas and associated cell signaling responses.

Significant amounts of proteins adsorbed on NPs are degraded within the lysosomes (e.g., serum albumin and transferrin) [132]. Still, some fragments remain for a long time within the lysosome lumen, and some smaller fragments are distributed widely within the cytosol [132]. The latter may induce alterations in cellular functionalities and contribute to the deregulation of cellular pathways [144,145,147,148]. A recent proteomics study revealed cell signaling pathways putatively affected by the protein corona [148]. Those pathways include oxidative stress response, mitochondrial energy metabolism, cell–cell contacts- and kinase-dependent signaling [148]. In the context of mTOR signaling, the protein corona may serve as an independent factor supporting mTOR activation [33]. However, studies that associate mTOR signaling with NPs’ protein coronas are at the very initial stage, and, so far, there is a lack of data on this issue [33,121,148,149].

Thus, there is a need for a systematic debugging of protein corona issues in general and to understand how protein coronas specifically affect mTOR signaling. Such progresses in the field will not only reduce the conflicts in nanotoxicology knowledge but also provide a fundamental basis for the use of nanomedicine approaches in the clinic.

## 6. Conclusions

In summary, it is difficult to decipher the mechanisms of mTOR activity modulation by NPs. In general, it is still not clear how exactly lysosomal pH and mTORC1 activation or inhibition are linked. The explanation of NP-induced lysosomal destabilization exclusively by the “proton sponge” hypothesis is not supported by most recent studies. We propose a tentative hypothesis outlined here, which still needs to be rigorously tested. Since only a few studies have used genetic/pharmacological blockade of mTOR signaling to directly confirm the involvement of mTOR in nanoparticle-mediated effects [25,116,122], we do not know yet all players involved in NP-mediated mTOR signaling. The role of the protein corona in this process awaits verification, and a more systematic approach is necessary to explore the mechanism by which nanomaterials interact with the mTOR pathway. The emerging picture points to lysosomes as key regulators of nanoparticle-induced signaling. It is essential to clarify how the whole cell adapts to nanoparticle engulfment. However, some progress in this area has now been achieved [150].

Regulators of the lysosome–mTORC1 pathway and its interplay with NP-induced signaling should also be investigated in more detail. The latter might be very important in mediating responses in cells exposed to subcytotoxic doses of NPs. Indeed, it has been shown recently that subcytotoxic doses of NPs induce specific morphological changes in cells [150,151]. Multiple studies have linked the disruption of organelles and other subcellular structures caused by NPs with cytotoxicity [13,152]. For instance, iron oxide NP-induced cytotoxicity is accompanied by oxidative stress, which is indicated by endogenous ROS production, lysosomal leakage, compromised mitochondrial potential and integrity, and mitochondrial substrate reduction [13,152,153]. It is worth noting that emerging studies indicate that mTOR modulates not only mitochondrial functions [154] but also mitochondrial dynamics [155].

The molecular knowledge of nanoparticle mediated mechanisms may be helpful in the treatment of malignancies. However, we have to deliberately assess the molecular foundations of NP–cell interactions. Thus, it is of great interest to further study the relationship between mTOR activity and lysosomal and mitochondrial dynamics and the perturbations induced by NPs.

## Figures and Tables

**Table 1:** Key types of nanoparticles (NPs) in approved NP-based medicines available for clinical use [7,8,80,82,85].

Type of Nanomaterials	Size Range	Clinical Indications
Polymer-based NPs	5 nm–5 µm	Severe combined immunodeficiency disease (SCID)
		Crohn’s disease
		Rheumatoid arthritis
		Psoriatic Arthritis
		Ankylosing Spondylitis
		Multiple Sclerosis (MS)
		Prostate Cancer
		Hepatitis B; Hepatitis C
		Acute lymphoblastic leukemia
		Chronic gout
		Hemophilia
Liposome formulations	≈100 nm	Pancreatic Cancer
		Fungal/protozoal infections
		Breast cancer
		Cutaneous T-Cell lymphoma
		Acute lymphoblastic leukemia
		Kaposi’s Sarcoma
		Ovarian cancer
		Fungal infections
Micellar NPs	10–200 nm	Antifungal
		Menopausal therapy
		Antineoplastic
		Aneasthesia
		Immunosuppressant
		Anti-HIV
Protein NPs	50–500 nm	Breast cancer
		Pancreatic cancer
		Cutaneous T-Cell lymphoma
Nanocrystals	50–1000 nm	Antiemetic
		Hyperlipidemia
		Immunosuppressant
		Anti-anorexic
		Psychostimulant
		Muscle relaxant
Inorganic and metallic NPs	10–200 nm	Glioblastoma
		Iron deficiency in chronic kidney disease
		Iron deficiency in patients undergoing chronic hemodialysis
		Iron deficiency anemia

**Table 2:** Effects of nanoparticles (NPs) on Mammalian target of rapamycin (mTOR) signaling.

NPs	Charge/Surface Modification	Size (nm)	Zeta Potential (mV)	Activity of mTOR	Ref.
**PS ^1^**	Positive/NH_2_	62 nm	+34.97 in dH_2_O −12.33 in DMEM	inhibited	[22]
**PS ^1^**	Positive/NH_2_	117 ± 17 nm	+54.4 in PBS	inhibited	[23]
**Iron oxide**	Negative/N.A. ^2^	51 nm	−39.3 in dH_2_O	inhibited	[24]
**Zinc oxide**	N.A. ^2^/N.A. ^2^	N.A.^2^	N.A. ^2^	inhibited	[111]
**PS ^1^**	Positive/NH_2_	30.6 ± 6.1 nm	+39.1 ± 6.5 in PBS	inhibited	[33]
**nano-TiO_2_**	N.A. ^2^/N.A. ^2^	21 nm	N.A. ^2^	inhibited	[112]
**UCNP** **Upconversion NPs**	Positive/poly-(allylamine hydrochloride) (PAH)	110 nm	+35 in PBS	inhibited	[113]
**SWCNT** **functionalized single-walled carbon nanotube**	N.A. ^2^/COOH	N.A. ^2^	N.A. ^2^	inhibited	[25]
**SWCNT** **functionalized single-walled carbon nanotube**	N.A. ^2^/N.A. ^2^	N.A. ^2^	N.A. ^2^	inhibited	[114]
**Silica**	N.A. ^2^/N.A. ^2^	62.1 ± 7.2 nm	−40 in dH_2_O	inhibited	[115]
**PAMAM** **polyamidoamine dendrimers**	N.A. ^2^/N.A. ^2^	N.A. ^2^	N.A. ^2^	inhibited	[116]
**Layered double hydroxide (LDH) NPs** **LDH-VP16 nanocomposites**	Positive/Etoposide (VP16)	105 nm	+39.9 in PBS	inhibited	[117]
**Bismuth NPs (BiNP)**	Negative/N.A. ^2^	63.72 nm in water 52.46 nm and 52.92 nm in PBS and DMEM	−27.43 ± 0.39 in dH_2_O −10.71 ± 0.53 in PBS −11.38 ± 0.5 in DMEM	inhibited	[118]
**Mesoporous silica NPs (MSNs)**	N.A. ^2^/BFA (Brefeldin A)	72 nm	N.A.	inhibited	[119]
**Multiwalled carbon nanotubes (MWCNTs)**	Negative/COOH	≈30–50 (outer), ≈5–12 (inner)	−30.5 ± 74.2 in ultrapure dH_2_O	inhibited	[120]
**Silica**	Positive/NH_2_	28.6 ± 4.2 nm	+36.9 ± 8.2 in PBS	activated	[33]
**Silica**	Negative/OH	31.2 ± 5.5 nm	−40.3 ± 7.4 in PBS	activated	[33]
**Copper Oxide**	Negative/N.A. ^2^	56.2 ± 22.9 nm in media 85.6 ± 27.2 nm in water	−0.057 in dH_2_O	activated	[121]
**Gold NPs**	N.A. ^2^/N.A. ^2^	2 nm	N.A. ^2^	activated	[122]
**Gold NPs**	N.A. ^2^/N.A. ^2^	30 nm	N.A. ^2^	activated	[123]
**PS**	Negative/COOH	119 ± 19 nm	−36.2 in PBS	activated	[23]

^1^ PS: Polystyrene; ^2^ N.A.: Not assessed; NH_2_: Amino group; COOH: Carboxyl group; OH: Hydroxyl group; mTOR: Mammalian/mechanistic target of rapamycin.
